# A case series on the role of ^18^F-FDG PET/CT-guided biopsy of osseous metastases

**DOI:** 10.1186/s41824-021-00095-1

**Published:** 2021-01-12

**Authors:** Michael Farrell, Gina Hyun, Michael P. Goold, Pavel Krapiva

**Affiliations:** Walter Reed National Military Medical Center, Bethesda, MD USA

**Keywords:** PET/CT, Bone biopsy, Osseous metastases

## Abstract

**Purpose:**

This case series explores the utility of positron emission tomography (PET)/computed tomography (CT) guidance for biopsy of ^18^F-fludeoxyglucose (FDG)-avid osseous lesions that are inconspicuous on CT.

**Methods:**

PET/CT-guided core biopsies were performed in four patients with suspected malignancies given ^18^F-FDG-avid osseous lesions that were inconspicuous on CT alone. The final diagnosis for each patient was determined by histopathological and molecular testing.

**Results:**

PET/CT-guided biopsy yielded accurate sampling via core needle biopsy (CNB) with histopathological confirmation of osseous metastases of the primary malignancy as opposed to a secondary malignancy in three patients and ruled-out metastatic spread in the fourth.

**Conclusion:**

PET/CT-guided biopsy of hypermetabolic osseous lesions that are inconspicuous on CT alone is an effective and safe diagnostic tool in patients with suspected malignancy.

**Supplementary Information:**

The online version contains supplementary material available at 10.1186/s41824-021-00095-1.

## Introduction

^18^F-FDG PET/CT is a noninvasive imaging modality that can be used for staging and monitoring treatment response among a variety of oncologic diseases. Studies report sensitivity and specificity for detecting most cancers between 84 and 100% and 83 and 100%, respectively (Nihayah et al., 2017).

This hybrid imaging technique combines the spatial resolution of CT with the metabolic differentiation of PET. Combining PET/CT imaging becomes of primary importance when targeting lesions either inconspicuous on CT or indistinguishable from an otherwise diffusely necrotic region. While other imaging modalities such as ultrasound and magnetic resonance imaging (MRI) have fulfilled similar roles in biopsy-guidance, these modalities are dependent upon the presence of structural differences between healthy and malignant tissues for successful sampling.

In one study, staging PET/CT discovered remote sites suspicious for a second, unrelated malignancy in 3–6% of patients with cancer, of which, one third were confirmed by biopsy as a new and separate primary malignancy (Klaeser et al., 2010). Biopsy is critical in determining the histologic origin of a morphologically or metabolically suspicious lesion. Histologic outcomes may include identification of benign tissue, metastasis of a known malignancy, or discovery of a new malignancy. Beyond its strengths in localizing target lesions, several studies have demonstrated the safety of PET/CT in guiding biopsies (Klaeser et al., 2010; Cornelis et al., 2014; El-Haddad, 2016). A safe and effective methodology for biopsies is critical for guiding clinical management.

## Materials and methods

### Patients

PET/CT-guided percutaneous core needle biopsies (CNB) were performed in four patients (three men and one woman ranging from 56 to 66 years of age with mean age 60.5) who had undergone whole body ^18^F-FDG PET/CT scans in the work-up of potentially metastatic disease. Only vertebral lesions with increased ^18^F-FDG uptake that were inconspicuous on CT were selected for inclusion. The final diagnoses, whether supporting or refuting osseous malignancies, were determined by histopathological and molecular testing analysis.

### ^18^F-FDG PET/CT-guided bone biopsy

All biopsies were performed under supervision of a board-certified radiologist and nuclear medicine physician in a PET/CT suite. Patients were confirmed to have fasted for at least 4 h prior to injection of ^18^F-FDG. The patients were placed in recumbent positions in darkened and quiet rooms during injection. Approximately 60 min after injection of radiotracer, the patients were positioned in the PET-CT scanner. A non-contrast low-dose CT scan was first obtained, centering on the target lesion. This was then followed by an emission scan of the same area. Images were obtained on a General Electric NM690 64-slice hybrid PET/CT using digital acquisition and time-of-flight reconstruction.

During biopsy, patients were positioned prone under sterile conditions after administration of conscious sedation and local anesthesia with 1% lidocaine. After the vertebral lesion of interest was identified on the initial PET/CT, additional CT images were obtained throughout the course of the biopsy needle placement followed by a second PET/CT acquisition to confirm accurate position prior to collecting the core sample. Generally, one sample was obtained per patient. A histopathology technologist on site was available to confirm the adequacy of the biopsy core.

## Case series

Patient 1 is a 66-year-old male with a history of chronic leukocytosis and 50 pack-year tobacco use who underwent routine virtual colonoscopy, which although negative for colonic neoplasia, did show a 7-cm right hilar mass, bilateral bulky adrenal glands, diffuse adenopathy, and ill-defined non-specific osseous hypodensities involving the sternum, L2, and L3 vertebral bodies. Three weeks after the initial CT, staging PET revealed ^18^F-FDG-avidity throughout the hilar mass, surrounding lymphadenopathy, manubrium, and L4 vertebral body. Areas of osseous photopenia previously identified amongst the L2 and L3 vertebral bodies were relatively void of ^18^F-FDG uptake. Subsequent transbronchial fine needle aspiration showed non-squamous non-small cell lung cancer with endocrine features (chromogranin and synaptophysin positive). Two weeks after staging, the patient underwent PET/CT-guided biopsy of a focal region of hypermetabolism involving the majority of the L4 vertebral body (Image 1). Biopsy indicated neuroendocrine carcinoma. Brain MRI showed 15–20 cystic foci of metastatic spread with midline shift and compression of the fourth ventricle. He clinically deteriorated with progressive ataxia and profound weakness, prompting initiation of whole brain irradiation followed by atezolizumab/carboplatin/etoposide. Despite some improvement after radiation and dexamethasone, he decompensated again with inability to eat or swallow pills and ceased returning to clinic for follow-up.

Patient 2 is a 57-year-old previously healthy male with a 35 pack-year smoking history who initially presented to primary care clinic with a week and a half of intermittent left shoulder pain radiating to his upper back awakening him from sleep. CT angiogram in the emergency department discovered diffuse lymphadenopathy with a possible lytic lesion within the T1 vertebral body. ^18^F-FDG PET/CT revealed stage IV metastatic disease affecting the cervical, supraclavicular, mediastinal, hilar, right internal mammary, retroperitoneal, and iliac lymph nodes. Numerous osseous sites including the entire sternum, T4, T10, right 7^th^ rib, and left iliac wing showed hypermetabolic activity. Spleen CNB showed large B cell lymphoma with indeterminate cell of origin. Initial bone marrow aspiration indicated no involvement; however, follow-up PET/CT-guided CNB of T10 (Image 2) showed CD10+ large B-cell lymphoma, guiding treatment with six cycles of R-CHOP. Mid-treatment PET/CT showed interval resolution of the majority of the previously seen hypermetabolic lesions with few foci of mild residual splenic and osseous hypermetabolism. Five months after initiation of chemotherapy, end-of-treatment PET/CT indicated complete response.

Patient 3 is a 56-year-old male with a long-standing history of gastroesophageal reflux disease and one pint of alcohol consumption daily who initially presented to gastroenterology clinic with 2 months of progressive dysphagia and mid-back pain. Endoscopic evaluation revealed esophageal adenocarcinoma with mucinous features and human epidermal growth receptor factor receptor 2 (HER2) positivity by immunohistochemistry. Subsequent CT demonstrated a few small, nonspecific upper lobe pulmonary nodules without obvious metastatic disease. Initial staging PET/CT confirmed ^18^F-FDG -avid mass at the gastroesophageal junction with regional lymphadenopathy. Numerous other hypermetabolic foci included subcentimeter left lower lobe pulmonary nodules, right hilar lymph node, mesenteric lymph nodes, descending colon, and multiple osseous lesions within the right humerus, right posterior iliac crest, T6, L3, and left lateral 2^nd^ rib. PET/CT-guided CNB biopsy of the T6 vertebral body (Image 3) revealed metastatic adenocarcinoma. Prior to end-of-therapy with FOLFOX and traztuzumab, the patient transferred care to an out-of-state medical facility, and no further information on the disease course is available.

Patient 4 is a 63-year-old female with a history of a verrucous mass along her left shin initially thought to be seborrheic keratosis temporarily improving with cryotherapy. After its recurrence 2 years later, she presented to dermatology clinic where shave biopsy revealed stage III melanoma with at least 1.8-mm infiltration and ulceration. A surgical oncologist performed wide local excision with sentinel lymph node biopsy, ultimately positive for malignant involvement. On staging PET/CT, inflammatory post-operative changes were identified along the anterior aspect of the left shin in addition to a hypermetabolic focus in the T9 vertebral body without correlating CT features. Further evaluation with MRI showed ill-defined focus of T9 vertebral bone marrow signal changes without discrete margins. PET/CT-guided biopsy of the ^18^F-FDG-avid region of the T9 vertebral body revealed no findings to suggest malignancy (Image 4). These findings correlated with a traumatic injury of her lumbar spine 5 years prior. Treatment with mivolumab was initiated, and surveillance imaging had not yet been obtained at the time of this review.

## Discussion

Since O’Sullivan et al. published the first study on the role of PET/CT as a diagnostic tool in musculoskeletal biopsy guidance in 2008, more studies have evaluated its efficacy in the diagnosis of a variety of metastases far beyond the scope of musculoskeletal lesions (Fei & Schuster, 2017; Cerci et al., 2016).

For both patients 1 and 2, CT identified non-specific osseous changes suggestive of but not specific for metastatic involvement. These sites of structural abnormalities failed to correlate with zones of ^18^F-FDG-avidity seen on PET/CT. In similar cases reported by Werner et al., multiple vertebral metastases were confirmed by repeat MRI and whole-body CT; however, a transpedicular biopsy of two separate vertebrae failed to produce conclusive histopathology beyond partially necrotic hematopoietic cells and trabecular bone (Werner et al., 2011). Such failed attempts may be avoided by PET/CT-guided biopsy of the focus with the highest metabolic activity.

As demonstrated by patient 3, various metastases may exhibit avid ^18^F-FDG uptake without structural abnormalities on MRI or CT. When obtaining such a biopsy, real-time visualization via PET/CT remains the most feasible imaging modality for guidance.

Patient 4 highlights the diagnostic specificity of PET/CT-guided biopsy. This patient’s past musculoskeletal trauma manifested as ^18^F-FDG-avidity on PET, which may have been mistaken for metastatic spread of melanoma if not for biopsy results supporting benign pathology.

## Conclusion

PET/CT is an invaluable imaging tool for the detection and staging of cancer through the mapping of hypermetabolic activity within the body. We demonstrated that PET/CT-guidance of vertebral lesions that are otherwise inconspicuous on CT alone is an effective, accurate, and safe method of biopsy. In three of the patients described in our case series, PET/CT-guided vertebral biopsy yielded confirmative results on first attempt. In a fourth patient, biopsy ruled-out osseous malignancy in an otherwise ^18^F-FDG -avid lesion. In conclusion, PET/CT-guided biopsy is an unparalleled diagnostic tool in the setting of osseous metastases as described in this case series.

## Supplementary Information

**Additional file 1.** CT Supplementary Images

## Data Availability

Data sharing is not applicable to this manuscript as no datasets were generated or analyzed during the series.
