# Supplementary material for: A case series on the role of 18F-FDG PET/CT-guided biopsy of osseous metastases
Source: Eur J Hybrid Imaging. 2021 Jan 12;5:1. doi: 10.1186/s41824-021-00095-1 (PMC8218035; doi:10.1186/s41824-021-00095-1)
Supplement: Supplementary file 1 — Additional file 1. CT Supplementary Images [file 41824_2021_95_MOESM1_ESM.pdf]

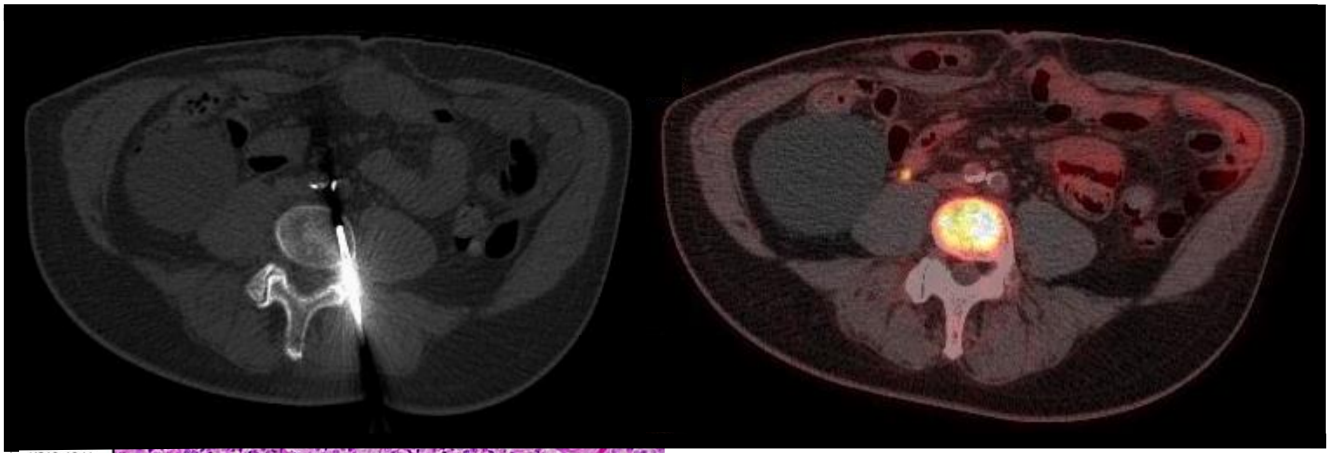

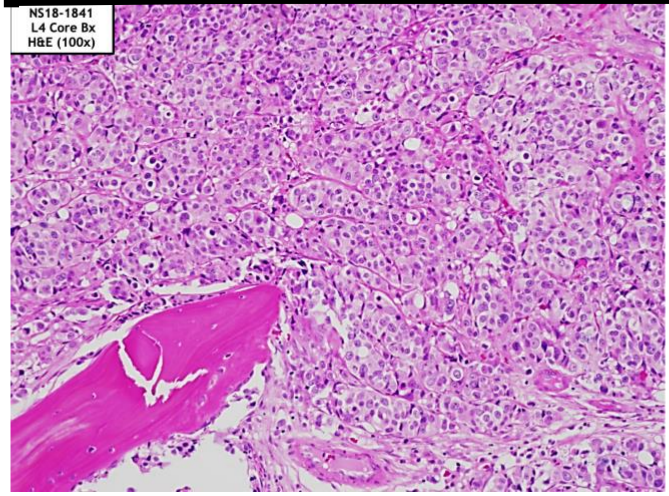

Image 1. Imaging and histopathology results from Patient 1. Lumbar CT axial view at the L4 vertebral level (top left) shows normal-appearing vertebral body intersected by core needle. PET/CT (top right) shows ^{18}F-FDG-avidity throughout the L4 vertebral body. At 100x, this H&E stain (bottom left) reveals nests of malignant cells replacing areas of bone marrow. The malignant cells contain moderate to abundant eosinophilic cytoplasm. The nuclei are round to oval with varied size and coarse speckled chromatin.

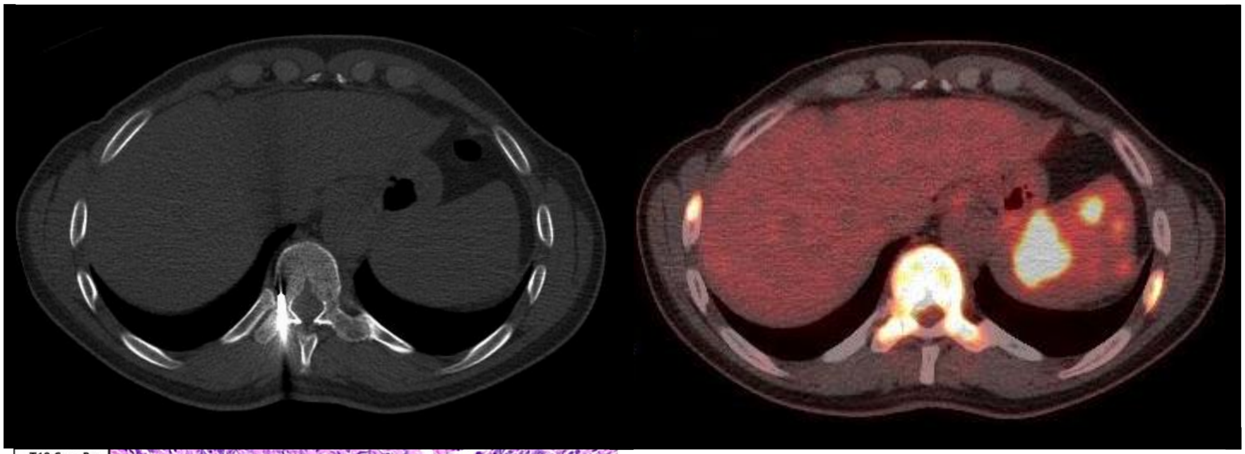

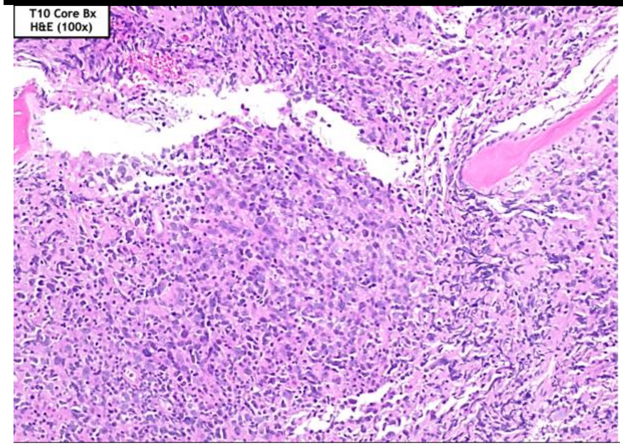

Image 2. Imaging and histopathology results from Patient 2. Thoracic CT with an axial view at the T10 vertebral level (top left) shows normal-appearing vertebral body intersected by core needle. PET/CT (top right) shows ^{18}F-FDG-avidity throughout the T10 vertebral body. H&E stain at 100x (bottom left) reveals bone marrow replaced by sheets of large neoplastic lymphocytes.

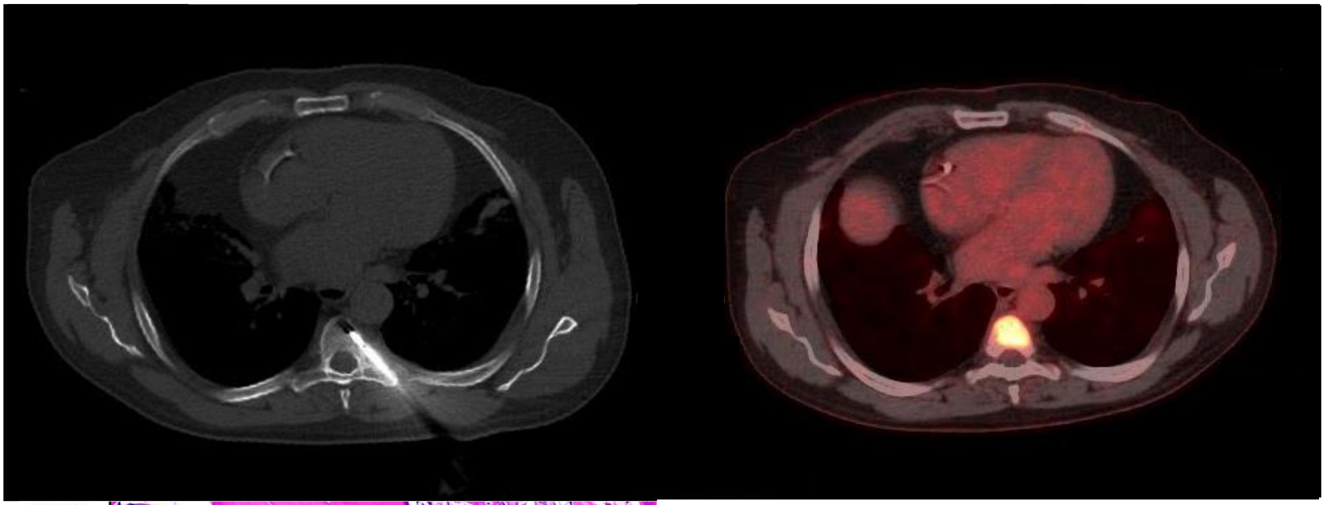

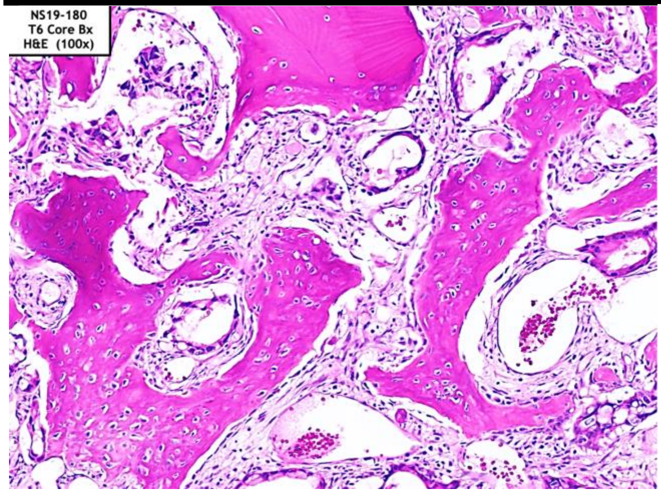

Image 3. Imaging and histopathology results from Patient 3. Thoracic CT axial view at the T6 vertebral level (top left) shows normal-appearing vertebral body intersected by core needle. PET/CT (top right) shows ^{18}F-FDG-avidity along the ventral T6 vertebral body. H&E stain at 100x (bottom left) reveals bone marrow diffusely replaced by a malignant cell line attempting to form glands.

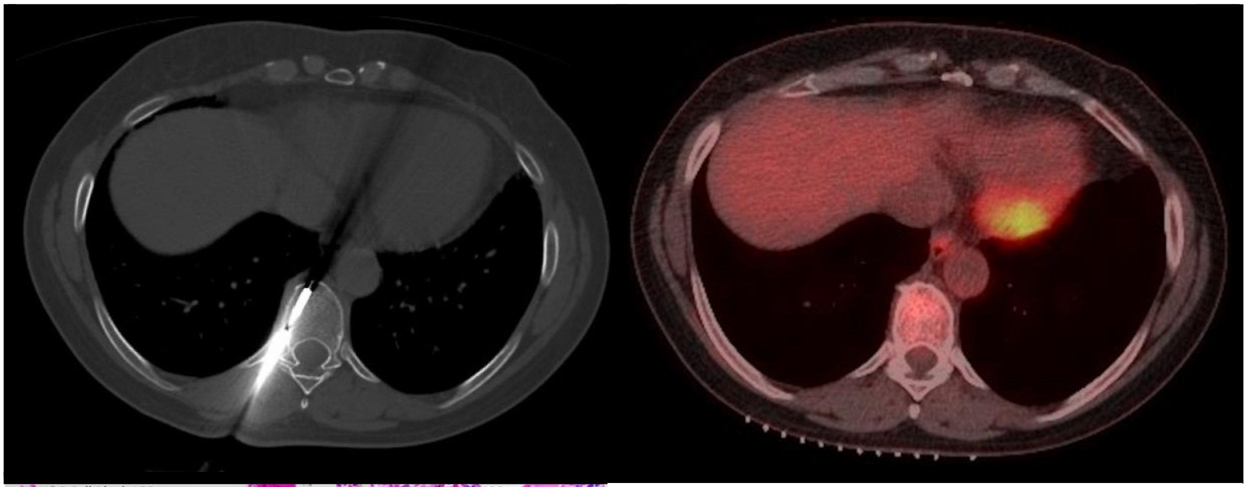

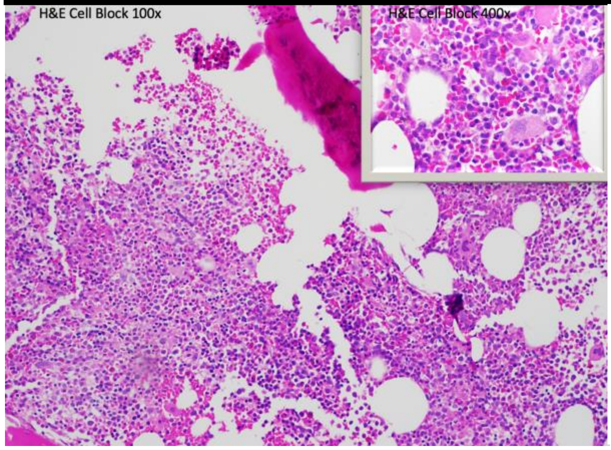

Image 4. Imaging and histopathology results from Patient 4. Thoracic CT axial view at the T9 vertebral level (top left) shows normal-appearing vertebral body intersected by core needle. PET/CT (top right) shows ^{18}F-FDG-avidity throughout the T9 vertebral body. H&E stain at 100x and 400x (bottom left) reveals normal trilineage hematopoietic cells. There were no dysplastic or neoplastic cells identified.
